# Dietary β-Glucan Alleviates Antibiotic-Associated Side Effects by Increasing the Levels of Antioxidant Enzyme Activities and Modifying Intestinal Microbiota in Pacific White Shrimp (*Litopenaeus vannamei*)

**DOI:** 10.3390/antiox13010052

**Published:** 2023-12-28

**Authors:** Yanbing Qiao, Fenglu Han, Xuhan Peng, Artur Rombenso, Erchao Li

**Affiliations:** 1 School of Life Sciences, East China Normal University, Shanghai 200241, China; 20071000110010@hainanu.edu.cn; 2 Key Laboratory of Tropical Hydrobiology and Biotechnology of Hainan Province, Hainan Aquaculture Breeding Engineering Research Center, School of Marine Biology and Fisheries, Hainan University, Haikou 570228, China; xhpeng@stu.xmu.edu.cn; 3 Commonwealth Scientific and Industrial Research Organisation (CSIRO), Agriculture and Food, Livestock & Aquaculture Program, Bribie Island Research Centre, Bribie Island, Brisbane, QLD 4507, Australia; artur.rombenso@csiro.au

**Keywords:** *Litopenaeus vanname*, antibiotics, β-glucan, antioxidant ability, intestinal microbiota

## Abstract

Antibiotics and their secondary metabolites are commonly found in aquatic ecosystems, leading to the passive exposure of many aquatic animals to low doses of antibiotics, which can affect their health. However, there is limited information available on how to mitigate the side effects of antibiotics on normal aquatic animals. This study aimed to investigate the potential of dietary β-glucan to alleviate the side effects induced by antibiotics in Pacific white shrimp (*Litopenaeus vannamei*) (0.37 ± 0.02 g). A six-week feeding trial was conducted with four dietary treatments including a control, 1 g/kg β-glucan (β-glucan), 50 mg/kg oxytetracycline (OTC), and a combination of 50 mg/kg OTC and 1 g/kg β-glucan (Mix) groups. At the end of the trial, the growth performance, intestinal microbial composition, antioxidant capacity, and immune response of the shrimp were assessed. There were no significant differences in growth performance among the groups, but the condition factor of the shrimp in the Mix group was significantly decreased when compared to the control and β-glucan groups. The activities of hepatopancreas catalase (CAT) and serum phenol oxidase in the OTC group were significantly lower than those in the control group. On the other hand, the activities of hepatopancreas superoxide dismutase and CAT enzymes in the β-glucan group were significantly higher than those in the OTC group. The supplementation of β-glucan in combination with antibiotics significantly increased the CAT activity and bacteriolytic activity compared to the OTC and control groups, respectively. Moreover, an analysis of the intestinal microbiota revealed that the Observed_species estimator in the Mix group was significantly higher than that in the control group. Dietary antibiotics significantly increased the abundance of Actinobacteria at the phylum level, but the Mix group showed no significant difference. The supplementation of β-glucan in combination with antibiotics also significantly increased the relative abundance of *Meridianimaribacter* compared to the control group. Additionally, the synergistic influence of β-glucan with antibiotics increased the beta diversity of intestinal microbiotas. These findings suggest that the supplementation of β-glucan in combination with antibiotics on Pacific white shrimp can alleviate the low antioxidant capacity and immune response caused by antibiotics while enhancing the intestinal microbial composition. This provides a potential solution to mitigate the negative impacts of antibiotics in aquaculture.

## 1. Introduction

Since their fortuitous discovery, antibiotics and related compounds have been extensively utilized in animal production for therapeutic diseases and as prophylactic agents [1,2]. However, the improper use, limited absorption in the body, and high solubility in water of antibiotics have resulted in increased antibiotic concentrations in the global aquatic environment [3], thereby causing adverse impacts on the aquatic ecosystem [4]. Consequently, there is growing concern regarding the potential effects of antibiotics, particularly in aquaculture.

Antibiotic stress can have a direct adverse impact on aquatic animals [5,6,7], such as Pacific white shrimp [8] and rainbow trout (*Oncorhynchus mykiss*) [9]. Moreover, antibiotic treatments can disrupt intestinal health, leading to reduced growth and immune function in aquatic animals [10,11]. The evidence indicates that the use of antibiotics in aquaculture should be strictly restricted due to the serious threat they pose to sustainability and the health of the ecosystem [12]. Additionally, only a limited number of antibacterial drugs have been approved for the treatment of bacterial diseases [11]. Oxytetracycline (OTC), a broad-spectrum tetracycline produced by *Streptomyces*, is one such drug [13]. Its mechanism of action involves interfering with bacterial protein synthesis [14]. OTC is widely used in global aquaculture practices due to its affordability and broad-spectrum activity [15,16], making it the go-to drug for treating bacterial diseases in aquaculture [17,18]. Research has shown that OTC is commonly used to treat bacterial infections, including vibriosis and Motile *Aeromonas* septicaemia [19]. However, some studies have reported that higher doses of OTC can induce immunosuppression in carp (*Cyprinus carpio* L.) [20]; affect oxidative stress and immunosuppression in rainbow trout [21,22], and hematopoiesis and osmoregulation in zebrafish (*Danio rerio*) and black tiger shrimp (*Penaeus monodon*) [23,24]; and even disturb the intestinal microbiome [23,25].

The gut system is a complex system consisting of various microbiota, which perform their functions through bacterial metabolism [26]. The diversity of intestinal microbial compositions is influenced by factors such as the environment, health conditions, and feed [27,28]. The structures and functions of the intestinal microbiota are closely linked to the physiological activity and health of the host [29]. Increasing evidence suggests that the administration of antimicrobial compounds disrupts the micro-ecological equilibrium of the intestinal microbiome and reduces the abundance and diversity of intestinal microbiota in various aquatic animals [30,31,32,33]. Moreover, conventional antibiotic treatment can disrupt the intestinal microbiome, which may have a negative impact on the health and welfare of farmed animals. Therefore, it is crucial to find ways to minimize the adverse influence on aquatic animals.

Dietary prebiotics have been found to have a positive impact on the host by enhancing non-specific immunity, regulating intestinal micro-organisms, and promoting the overall health of the host [34,35,36]. β-(1,3)-Glucan, a natural polymer derived from the cell wall of yeast and mold, is known to be one of the most effective immunostimulants [37]. It has been shown to activate the expression of immune-related genes [38]. Several studies have demonstrated that β-glucan can enhance the immunity of aquatic animals and improve their resistance to diseases by increasing immunostimulants [39,40,41]. In addition, dietary β-glucan has been found to improve the structure of the intestinal microbial community and promote the growth of beneficial bacteria in Pacific white shrimp [42]. Post-antibiotic probiotic supplementation has also been shown to restore the structure of the intestinal microbial community [43]. However, the potential of adding β-glucan to mitigate the side effects caused by dietary antibiotics is still not well-understood, particularly in terms of its effects on growth performance, intestinal microbial composition, antioxidant capacity, and non-specific immunity.

Aquaculture has experienced significant growth over the past seventy years and has become a prominent source of protein and nutrition for human consumption [44]. Among the various species farmed in aquatic environments, the Pacific white shrimp has emerged as the dominant crustacean species, accounting for 52.9% of total farmed crustacean production in 2019 [45]. The shrimp-farming industry has witnessed tremendous development and has generated substantial economic benefits. Therefore, the objective of this study was to assess the potential of β-glucan supplementation in mitigating the adverse effects of dietary antibiotics on growth performance, intestinal microbial composition, antioxidant capacity, and immune response in *Litopenaeus vannamei*.

## 2. Materials and Methods

### 2.1. Dietary Design

Four isonitrogen and isolipidic practical diets were formulated for the experiment. The diets contained β-(1,3)-glucan at a concentration of 1 g/kg [42] and OTC at a concentration of 50 mg/kg [46]. These additions were incorporated into the control diet, which contained at least 35% protein and at least 7% lipid (Table 1). The experiment included four groups: a group fed the control diet (Control), a group fed the control diet with 1 g/kg β-glucan (β-glucan), a group fed the control diet with 50 mg/kg OTC, and a group fed the control diet with both 50 mg/kg OTC and 1 g/kg β-glucan (Mix). The diets were manufactured following the procedures described in our previous study [42]. In summary, the ingredients were crushed (80 m) and thoroughly mixed, and then distilled water was added to form a stiff dough. The diets were then press-pelleted using a 2 mm die (F-26, SCUT, industrial factory, Guangdong, China). After drying at 37 °C in the absence of light, the diets were manually ground, sieved, and stored in plastic containers at −20 °C until use. The diets were analyzed for crude protein, total lipid, and crude ash using the methods outlined by AOAC [47]. The proximate composition of the diets is presented in Table 1.

### 2.2. Experimental Shrimp and Design

Pacific white shrimp (PL 5) were obtained from Blue Ocean Biotechnology Co., Ltd., a shrimp farm located in Wenchang, Hainan, China. Upon arrival at the laboratory, the shrimp were cultured in normal salinity conditions and acclimatized for two weeks in a 500 L polyethylene tank. During this period, the larvae were fed commercial feed (Alpha Feed Co., Ltd. (Shenzhen, China), Protein 48%) four times a day at 4% of their larval weight to meet their nutritional needs. After acclimatization, shrimp weighing 0.37 ± 0.02 g were randomly selected and stocked into sixteen tanks (60 × 35 × 40 cm) at a density of 20 shrimp per tank. Each group had four replicates. Over the six-week experimental period, the shrimp were hand-fed the experimental diets four times daily (at 07:00, 12:00, 17:00, and 24:00) at a rate of 6–8% of their body weight. Water quality conditions and daily management procedures were conducted as previously described [42].

### 2.3. Sample Collection and Calculation

After completing the feeding experiments, the shrimp were subjected to a 24 h period of starvation. Following this, they were anesthetized by exposure to shattered ice. The number, weight, and body length of each shrimp in each tank were measured to determine their survival, specific growth rate, and weight gain. From each tank, fourteen shrimp were randomly selected, and hemolymph was collected from their abdomens using sterile 1 mL syringes. The collected serum was stored overnight at 4 °C, and the supernatant was obtained by centrifugation (at 4 °C, 3500× *g* for 10 min) and stored at −80 °C for further analysis. The hepatopancreas and mid-intestine were dissected from the shrimp using sterile techniques (after hemolymph collection). The weight of each hepatopancreas was measured to calculate the hepatosomatic index, while the hepatopancreas and serum were analyzed for antioxidant and immune enzyme analysis, respectively. The mid-intestine was analyzed for microbiota. All samples were immediately placed in freezer tubes and stored at −80 °C until further analysis. Growth performance related parameters were calculated including the following:

Growth data were calculated using the following formulae:

Weight gain (%) = (final weight − initial weight)/initial weight × 100

Specific growth rate (% day^−1^) = (ln final weight − ln initial weight)/day × 100

Condition factor (%) = final weight (g)/(final body length, cm)^3^ × 100

Hepatosomatic index (%) = hepatosomatic weight/final weight × 100

### 2.4. Immune and Antioxidant Enzyme Activities

The two hepatopancreas samples in each tank weighing 1 g were accurately weighed and homogenized in a cold 0.86 saline solution (1:9, *w*/*v*) using a tissue grinder, and eight in each group were analyzed. The homogenate was centrifuged at 4 °C for 10 min at 2500× *g*, and the resulting supernatant was used for antioxidant enzyme activity analysis. These hepatopancreas samples were used to measure the activities of total protein using the coomassie brilliant blue method (code: A045-2-2), superoxide dismutase (SOD) using the WST-1 method (code: A001-3-2), catalase (CAT) using the ammonium molybdate spectrophotometric method (code: A007-1-1), and glutathione peroxidase (GPX) using a colorimetric method (code: A005-1-2). The malondialdehyde (MDA) content was measured using the TBA method (code: A003-1-2) with commercial kits (Nanjing Jiancheng Institute, Nanjing, China). The enzymatic activities were determined according to previously established procedures [48,49]. The two serum samples from each tank were randomly obtained for immune enzyme activity analysis. Phenol oxidase (PO) activity in the serum was measured following the method described by Hernández-López [50]. Bacteriolytic activity in serum was as described by Hultmark [51].

### 2.5. Immune and Antioxidant Enzyme Activities

The three gut samples, one from each tank, were individually obtained for analyzing the gut microbiota. Intestinal microbiota analysis was performed by Majorbio Biopharm Technology Co., Ltd. (Shanghai, China). All intestinal samples were subjected to total genomic DNA extraction using the TAB/SDS method, and the quality of the extracted DNA was evaluated using a NanoDrop spectrophotometer (Thermo, Wilmington, DE, USA). Next-generation sequencing was carried out on an Illumina HiSeq platform following the protocol provided by Majorbio Biopharm Technology Co., Ltd. (Shanghai, China). The sequencing process and analysis of results followed a previous procedure [42]. The libraries were ultimately sequenced on an Illumina HiSeq 2500 platform, and all analyses were performed using R package software (version 4.0.2, https://www.R-project.org). The alpha diversity index values were expressed as the mean ± SE and analyzed using two-way ANOVA (SPSS 23.0), followed by multiple comparisons using Duncan’s test. The statistically significant differences were set at *p* < 0.05. The sequences were deposited in the NCBI Sequence Read Archive (SRA) under accession number PRJNA843590.

### 2.6. Statistical Analysis

All experimental results were analyzed using IBM SPSS 22.0 (Armonk, NY, USA), and data were analyzed for homoscedasticity and graphed using GraphPad Prism 8 (Boston, MA, USA). The data were presented as the mean ± standard error (SE) (*n* = 4). One-way analysis of variance (ANOVA) was used for comparisons. The statistically significant differences were set at *p* < 0.05.

## 3. Results

### 3.1. Growth Performance

**Figure 1:**
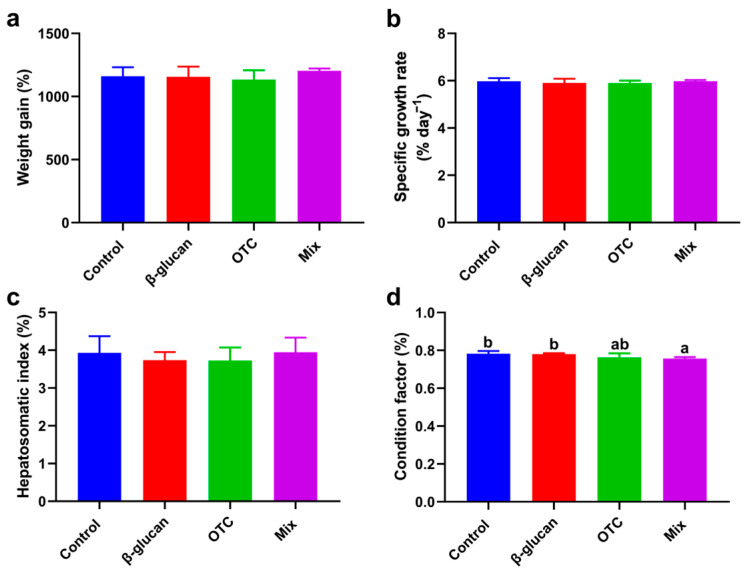
Effects of β-glucan protect *Litopenaeus vannamei* from antibiotic-induced side effects on growth performance: (**a**) weight gain, (**b**) specific growth rate, (**c**) hepatosomatic index, and (**d**) condition factor. All data are expressed as the mean ± SE (*n* = 4). Bars with different letters represent significant differences (*p* < 0.05) among groups.

The results showed that there were no significant differences in weight gain, specific growth rate, and hepatosomatic index between the control group and the other experimental groups (*p* > 0.05, Figure 1a–c). Shrimp fed the Mix diet had a lower condition factor than that in the control and β-glucan groups (*p* < 0.05, Figure 1d). Although a decline in the condition factor was also observed in the OTC group compared with the control and β-glucan groups, the difference was not significant (*p* > 0.05).

### 3.2. Antioxidant and Immune Capacity

**Figure 2:**
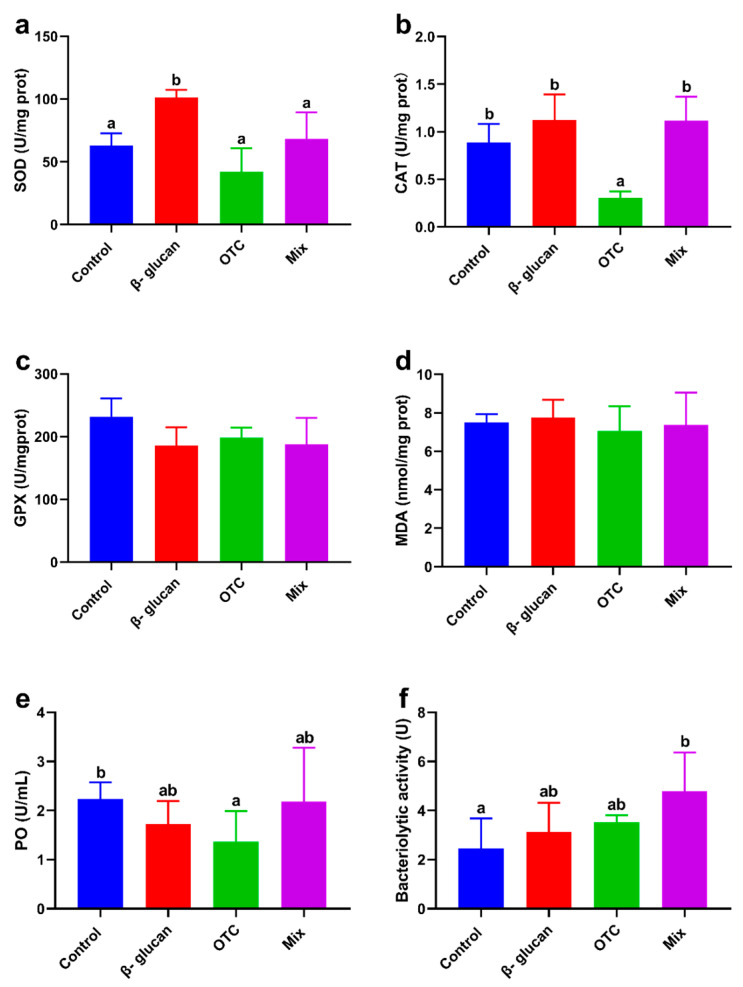
Effects of β-glucan protect *Litopenaeus vannamei* from antibiotic-induced side effects on antioxidant and immune capacity: (**a**) superoxide dismutase (SOD) activity (hepatopancreas), (**b**) catalase (CAT) activity (hepatopancreas), (**c**) glutathione peroxidase (GPX) activity (hepatopancreas), (**d**) malondialdehyde (MDA) content (hepatopancreas), (**e**) phenol oxidase (PO) activity (serum), and (**f**) bacteriolytic activity (serum). Bars with different letters represent significant differences (*p* < 0.05) among groups.

The activity of SOD was significantly increased in the β-glucan group compared to the other groups (*p* < 0.05, Figure 2a). Conversely, the activity of CAT was significantly decreased in the OTC group compared to the other groups (*p* < 0.05, Figure 2b). The serum of shrimp fed the OTC diet showed significantly lower PO activity compared to the control group (*p* < 0.05, Figure 2e). Furthermore, the Mix group exhibited significantly higher serum bacteriolytic activity than the control group (*p* < 0.05, Figure 2f). However, there were no significant differences in GPX activity and MDA content in the hepatopancreas among the groups (*p* > 0.05, Figure 2c,d).

### 3.3. Composition and Diversity of the Intestinal Microbiota

**Figure 3:**
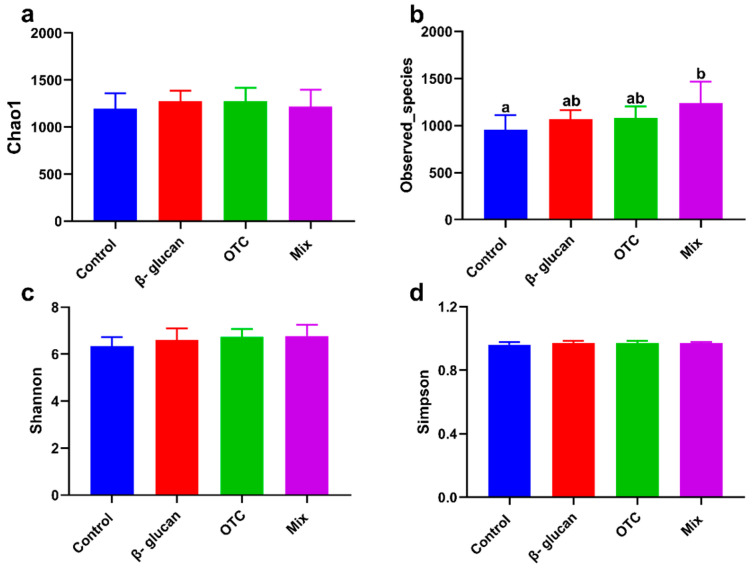
Effects of β-glucan protect *Litopenaeus vannamei* from antibiotics-induced side effects on the alpha diversity of intestinal microbiota: (**a**) Chao1 estimator, (**b**) Observed_species estimator, (**c**) Shannon estimator, and (**d**) Simpson estimator. Bars with different letters represent significant differences (*p* < 0.05) among groups.

**Figure 4:**
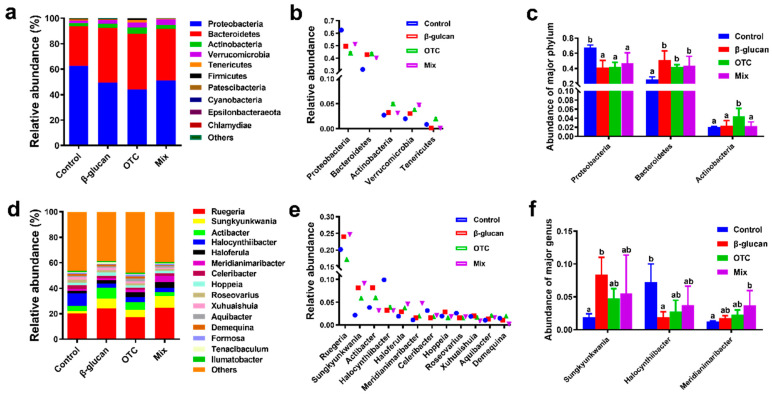
Effects of β-glucan protect *Litopenaeus vannamei* from antibiotics-induced side effects at the phylum and order level: (**a**) Microbiota composition at the phylum level with relative abundance in the top four. (**b**) Relative abundance of gut microbiota at the phylum level. (**c**) Differences in the relative abundance of phylum taxa among groups. (**d**) Microbiota composition at the order level with relative abundance in the top ten. (**e**) Relative abundance of intestinal microbiota at the order level. (**f**) Differences in the relative abundance of order taxa among groups. Bars with different letters represent significant differences (*p* < 0.05) among groups.

**Figure 5:**
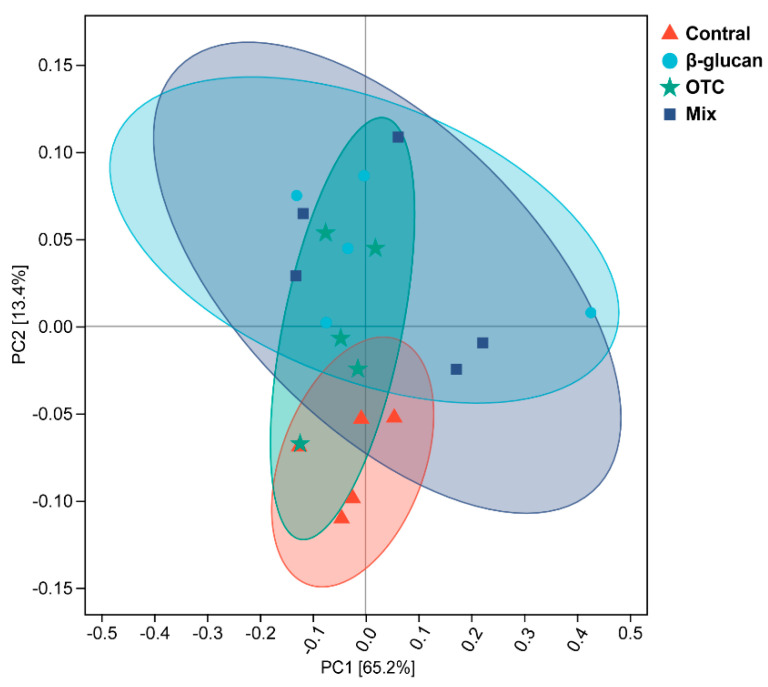
Effects of β-glucan protect *Litopenaeus vannamei* from antibiotic-induced side effects on the beta diversity of intestinal microbiota. PCoA of the microbiota at the OTU level is based on Jaccard distances. Analysis of similarity (ANOSIM) tests were performed to evaluate the overall differences in bacterial community structure based on Jaccard distance.

A total of 1,732,508 clean sequences, with an average length of 86,625 bp, were obtained from the intestinal contents, with lengths ranging from 108 to 432 bp. The effects of β-glucan on protecting shrimp from antibiotic-induced side effects on the intestinal alpha diversity indices of shrimp were shown in Figure 3. Compared with the control group, the Observed_species index was significantly increased in the Mix group (*p* < 0.05, Figure 3b). However, there were no significant differences in the Chao1, ACE, and Shannon indices among the groups (*p* > 0.05). At the phylum level, the effects of β-glucan protecting shrimp from antibiotic-induced side effects on the intestinal microbiota are shown in Figure 4. The most dominant phyla (%) in all samples were Proteobacteria, Bacteroidetes, Actinobacteria, and Verrucomicrobia (Figure 4a,b). Compared with the control group, the abundance of Proteobacteria significantly decreased in the other groups (*p* < 0.05). Conversely, the abundance of Bacteroidetes increased in all experimental groups (*p* < 0.05). The relative abundance of Actinobacteria in the OTC group was significantly higher than that in the other groups (*p* < 0.05). At the order level, the effects of β-glucan and OTC on the intestinal microbiota of shrimp were shown in Figure 4. The relative abundance of *Meridianimaribacter* in the Mix group was significantly increased compared to that in the control group (*p* < 0.05). Compared with the control group, the relative abundance of *Sungkyumkwania* was significantly increased in the additional β-glucan group, while the relative abundance of *Halocynthibacter* showed the opposite trend. Furthermore, the beta diversity analysis between different experimental groups based on a PCA plot is shown in Figure 5. Samples in all additional β-glucan groups had similar components. The intestinal microbiota structure of shrimp in the control group was significantly different from the other groups. Moreover, additional OTC also significantly affected the intestinal microbiota structure compared to β-glucan groups.

## 4. Discussion

In recent decades, the high stocking densities in aquaculture have made infectious diseases a significant threat [52]. This has led to the widespread misuse and overuse of antibiotics, particularly in low- and middle-income countries [53,54,55]. Consequently, both aquatic animals and their environment have faced serious problems. To counter the invasion of external bacteria, the host’s immune system plays a crucial role. While antibiotic-like substances may benefit harmful bacteria [56], their long-term use can negatively impact the normal physiological functions of host cells [57] and even cause destructive effects [58]. In recent years, extensive research has been conducted on β-glucans as potential immunoadjuvants [59]. However, there is limited research on whether β-glucan can protect Pacific white shrimp from antibiotic-induced side effects, including those affecting intestinal microbiota. Therefore, this study aims to investigate whether the addition of β-glucan can improve the side effects of antibiotics, such as antioxidant and immune capacity, as well as intestinal microbiota structures.

The hepatopancreas is a crucial organ involved in crustacean molting and plays a vital role in energy storage and breakdown, nutrient accumulation, and carbohydrate and lipid metabolism [60,61]. Long-term exposure to low-level antibiotic use has been found to induce growth performance in aquatic animals such as rainbow trout [22] and sea cucumber (*Apostichopus japonicus selenka*) [62]. Dietary β-glucan has also been shown to enhance growth performance in rainbow trout and Pacific white shrimp [63,64]. However, this study suggests that the long-term addition of β-glucan and OTC at legal doses restrains the growth performance (condition factor) in juvenile shrimp, and additional OTC could further decrease the condition factor. This result aligns with a previous study where the condition factor decreased when antibiotics were added to zebrafish during the growth period [65]. Nile tilapia (*Oreochromis niloticus*) exposed to antibiotics exhibited a lower hepatosomatic index compared to normal fish [10]. A 14-day dietary OTC treatment at a concentration of 18 g kg^−1^ had no effect on the growth performance of shrimp [66]. The levels of antibiotics in the hepatopancreas can remain higher for a longer period of time compared to the muscle [67]. Based on these findings, it is postulated that antibiotics can hinder the nutritive accumulation in the hepatopancreas, leading to atrophy and redirecting extra energy to mitigate the side effects of the antibiotic.

The hepatopancreas plays a crucial role in the antioxidant capacity of shrimp [68]. Specifically, it produces important antioxidant enzymes such as SOD, CAT, and GPX enzymes in Pacific white shrimp. These enzymes are essential for maintaining overall health and reducing oxidative-stress-induced damage to the hepatopancreas [69,70]. SOD acts as an oxidoreductase that converts superoxide radicals into H_2_O_2_ and molecular oxygen, thereby protecting cells from their harmful effects [71]. Catalase works synergistically with SOD by efficiently utilizing hydrogen peroxide and clarifying active oxygen free radicals in organisms [72]. In the field of aquaculture, antibiotics have been commonly used to treat bacterial diseases in farmed animals [73,74]. However, the indiscriminate use of antibiotics can negatively impact normal physiological functions and disturb antioxidant capacity [75]. This research observed that the long-term oral administration of OTC at legal doses reduced the activity of SOD and CAT enzymes, but the addition of β-glucan with OTC increased their activities. Similar findings have been reported in rainbow trout, where dietary antibiotics induced antioxidant enzymes [21]. Additionally, the administration of sulfamethoxazole and OTC decreased SOD and CAT enzyme activity in the intestine of the oriental river prawn (*Macrobrachium nipponense*) [76]. These results indicate that antibiotics used in aquaculture animals have a detrimental effect on antioxidant capacity. On the other hand, β-glucan has been shown to enhance the resistance to pathogens in common carp (*Cyprinus carpio*) [77] and increase the antioxidant capacity in Pacific white shrimp [42]. In summary, dietary β-glucan can enhance antioxidant capacity and provide partial protection against OTC-induced low antioxidant defenses in shrimp.

Numerous studies have documented the immunomodulatory effect of antibiotics on aquatic animals [24]. Shrimp, as invertebrates, rely on activating innate immune responses to combat pathogens through immunization and defense systems [78]. These systems include humoral and cellular mechanisms [79], with immune proteins such as lysozyme and prophenoloxidase playing a crucial role [78,80]. Prophenoloxidase (PO) in crustaceans is activated by invading microbes and is directly related to the animals’ immune condition, as it is involved in the defense reaction [81,82]. Antibiotics can affect the nonspecific immunity of shrimp, leading to a decrease in PO activity in shrimp hemocytes when OTC is added [83]. In a study with black tiger shrimp, the PO activity was significantly reduced after injection with OTC for 3–7 days [24]. Lysozymes, which hydrolyze bacteria and contribute to animal digestion and innate immunity, have been found to have increased bacteriolytic activity when OTC and β-glucan are added to shrimp, indicating the synergistic effects of β-glucan and OTC, consistent with previous research on hybrid giant tiger groupers (*Epinephelus fuscoguttatus* × *Epinephelus lanceolatus*) and Nile tilapia [84,85]. These findings suggest that the dietary addition of β-glucan, with or without OTC, could enhance the immune capacities of shrimp.

Antibiotics have the ability to kill harmful bacteria, but prolonged use can result in the elimination of normal or beneficial microbiota and an increase in resistance in aquatic animals [33]. Various studies have demonstrated that additional antibiotics can alter the composition of the intestinal microbiome at the phylum and order levels in aquatic organisms [86]. The administration of over-the-counter (OTC) antibiotics can significantly modify the bacterial composition [76,87]. However, empirical research has shown that the addition of prebiotics can support the intestinal microbiota community [34,88]. In this study, the combination of β-glucan and OTC significantly increased the α-diversity of intestinal micro-organisms, indicating the synergistic effects of β-glucan and OTC. Previous research has also indicated that oral OTC can impact the composition of intestinal micro-organisms [86]. In this study, the Proteobacteria was the dominant phylum in all groups, consistent with other research [89]. Actinobacteria, which are associated with intestinal inflammation, have been identified in both humans and animals [90]. However, this study observed a significant increase in the relative abundance of Actinobacteria in the OTC group compared to the other groups. Previous literature has suggested that such upregulation can be harmful to hosts, as many bacteria in this phylum were called opportunistic pathogens [91]. In contrast, few genera in Actinobacteria were reported to be probiotics in aquaculture [92]. This research suggest that antibiotics may promote the growth of Actinobacteria, but the addition of β-glucan can ameliorate this change. The order *Meridianimaribacter* is a Gram-negative and rod-shaped order of bacteria from the family *Flavobacteriaceae*. It can produce extracellular cellulases and hemicellulose, contributing to digestion [93]. In this study, combining β-glucan and OTC increased the abundance of the order *Meridianimaribacter* in the intestinal contents. Studies have suggested that such a combination addition can promote nutrition metabolism and, thereby, restrain the overgrowth of facultative bacteria. The good health of shrimp is related to the structure and function of intestinal micro-organisms [94]. Additionally, the addition of probiotic substances can positively impact the restoration of normal microbial diversity in the intestine [95]. This study demonstrated that oral antibiotics reduced microbial diversity in shrimp, but the addition of β-glucan helped restore it. Similar results were observed in black tiger shrimp [66], and another study showed that β-glucan could improve bacterial diversity in shrimp [42]. Therefore, it is speculated that supplementing β-glucan in combination with antibiotics can establish a more diversified and stable microbiota, and potentially rebuild the intestinal microflora.

## 5. Conclusions

This research aimed to investigate the impact of dietary OTC on the antioxidant capacity, nonspecific immune system, and intestinal microbiota in Pacific white shrimp. The findings revealed that dietary OTC reduced the antioxidant capacity and suppressed the nonspecific immune system. Moreover, it also influenced the composition and structure of the intestinal microbiota. However, the addition of β-glucan to the diet increased the antioxidant capacity, prevented nonspecific immune responses, and restored the intestinal microflora that were affected by OTC. Therefore, administering β-glucan could offer partial protection to Pacific white shrimp (*Litopenaeus vannamei*) from the side effects of antibiotics, both in terms of physiological response and intestinal microbial composition. This research provides valuable insights for developing effective strategies to enhance aquatic health and maintain a healthy intestinal microbiota. However, further research is needed to fully understand the molecular mechanisms underlying the protective effects of β-glucan against the side effects of OTC.

## Figures and Tables

**Table 1:** Formulation and proximate composition of the experimental diets (% dry matter).

Ingredients	Dietary β-(1,3)-Glucan Concentration			
	Control	β-Glucan	OTC	Mix
Fish meal (g)	26	26	26	26
Soybean meal (g)	28	28	28	28
Corn starch (g)	23	23	23	23
Shrimp meal (g)	4	4	4	4
Calcium dihydrogen phosphate (g)	1.5	1.5	1.5	1.5
Vitamin premix ^1^ (g)	2	2	2	2
Mineral premix ^2^ (g)	2	2	2	2
Choline chloride (g)	1	1	1	1
Fish oil (g)	2.5	2.5	2.5	2.5
Soybean oil (g)	2.5	2.5	2.5	2.5
Soybean lecithin (g)	1	1	1	1
Cholesterol (g)	0.5	0.5	0.5	0.5
Carboxymethylcellulose (g)	3	3	3	3
Butylated hydroxytoluene (g)	0.1	0.1	0.1	0.1
Microcrystalline cellulose (g)	2.9	2.8	2.895	2.795
β-(1,3)-Glucan ^3^ (g)	0	0.1	0	0.1
Oxytetracycline ^4^ (g)	0	0	0.005	0.005
Total	100	100	100	100
Nutrient levels (%)				
Crude protein	35.2	35.5	35.3	35.5
Total lipid	7.6	7.6	7.6	7.6
Ash	10.3	10.5	10.6	10.7
Moisture	9.2	9.2	9.2	9.2

^1^ Vitamin premix (per kg of diet): vitamin A: 4800 IU; L-ascorbyl-2-polyphosphate: 35%; Active C: 35.71 g; folic acid: 0.18 g; biotin: 0.05 g; riboflavin 3 g; DL Ca-pantothenate L: 5 g; pyridoxine HCl B_6_: 1 g; vitamin B_12_: 0.002 g; thiamine HCl: 0.5 g; Menadione K_3_: 2 g; DL-alpha-tocopheryl acetate: 20 IU; inositol: 5 g; nicotinamide: 5 g; vitamin D: 8000 IU. ^2^ Mineral premix (per kg of diet): ZnSO_4_·H_2_O: 20.585 g; Ca (IO_3_)_2_: 0.117 g; CuSO_4_·5H_2_O: 0.625 g; MnSO_4_ H_2_O: 1.625 g; MgSO_4_·H_2_O: 39.86 g; CoCl_2_: 0.01 g; FeSO_4_·H_2_O: 11.179 g; CaHPO_4_·2H_2_O: 166.442 g. ^3^ Yeast β-glucan was purchased from Xi’an Ruilin Biotechnology Co., Ltd., Xi’an, China. ^4^ Oxytetracycline (OTC) was purchased from Beijing Solaibao Technology Co., Ltd., Beijing, China.

## Data Availability

The data of 16S rRNA sequence presented in the study are deposited in https://www.ncbi.nlm.nih.gov/sra (accessed on 1 December 2022), under accession number PRJNA843590.
